# Design and Organization of the Dexamethasone, Light Anesthesia and Tight Glucose Control (DeLiT) Trial: a factorial trial evaluating the effects of corticosteroids, glucose control, and depth-of-anesthesia on perioperative inflammation and morbidity from major non-cardiac surgery

**DOI:** 10.1186/1471-2253-10-11

**Published:** 2010-06-30

**Authors:** Basem Abdelmalak, Ankit Maheshwari, Edward Mascha, Sunita Srivastava, Theodore Marks, WH Wilson Tang, Andrea Kurz, Daniel I Sessler

**Affiliations:** 1 Departments of General Anesthesiology and OUTCOMES RESEARCH, Cleveland Clinic, Cleveland, Ohio, USA; 2 Anesthesiology Institute. Cleveland Clinic, Cleveland, Ohio, USA; 3 Departments of Quantitative Health Sciences and OUTCOMES RESEARCH. Cleveland Clinic, Cleveland, Ohio, USA; 4 Vascular Surgery. Cleveland Clinic, Cleveland, Ohio, USA; 5 Department of General Anesthesiology. Cleveland Clinic, Cleveland, Ohio, USA; 6 Heart and Vascular Institute, Cleveland Clinic, Cleveland, Ohio, USA; 7 Department of OUTCOMES RESEARCH. Cleveland Clinic, Cleveland, Ohio, USA; 8 Department of Anesthesia, McMaster University, Hamilton, Ontario, Canada

## Abstract

**Background:**

The perioperative period is characterized by an intense inflammatory response. Perioperative inflammation promotes postoperative morbidity and increases mortality. Blunting the inflammatory response to surgical trauma might thus improve perioperative outcomes. We are studying three interventions that potentially modulate perioperative inflammation: corticosteroids, tight glucose control, and light anesthesia.

**Methods/Design:**

The DeLiT Trial is a factorial randomized single-center trial of dexamethasone vs placebo, intraoperative tight vs. conventional glucose control, and light vs deep anesthesia in patients undergoing major non-cardiac surgery. Anesthetic depth will be estimated with Bispectral Index (BIS) monitoring (Aspect medical, Newton, MA). The primary outcome is a composite of major postoperative morbidity including myocardial infarction, stroke, sepsis, and 30-day mortality. C-reactive protein, a measure of the inflammatory response, will be evaluated as a secondary outcome. One-year all-cause mortality as well as post-operative delirium will be additional secondary outcomes. We will enroll up to 970 patients which will provide 90% power to detect a 40% reduction in the primary outcome, including interim analyses for efficacy and futility at 25%, 50% and 75% enrollment.

**Discussion:**

The DeLiT trial started in February 2007. We expect to reach our second interim analysis point in 2010. This large randomized controlled trial will provide a reliable assessment of the effects of corticosteroids, glucose control, and depth-of-anesthesia on perioperative inflammation and morbidity from major non-cardiac surgery. The factorial design will enable us to simultaneously study the effects of the three interventions in the same population, both individually and in different combinations. Such a design is an economically efficient way to study the three interventions in one clinical trial vs three.

**Trial registration:**

**This trial is registered at **Clinicaltrials.gov **#**: NTC00433251

## Background

Mortality remains substantial among high-risk patients (ASA physical status 3-5)[1]. In some populations, it is estimated to be as high as 5-14% during the first postoperative year[2-4]. Efforts to identify interventions that decrease perioperative morbidity and mortality are thus warranted. The perioperative period is characterized by an intense inflammatory response marked by elevated concentrations of inflammatory markers like C-Reactive Protein (CRP). Inflammation is the body's response to tissue injury[5] and results in production of inflammatory mediators[6], which are associated with considerable postoperative morbidity. For example, cardiovascular mortality is responsible for 17% of post-operative deaths[3]. Inflammation has been an important mediator in the pathophysiology of myocardial infarction, at all stages in the development of vulnerable plaque, from initial lipid deposition to plaque rupture. Thus, markers of systemic inflammation such as CRP may better predict perioperative cardiac morbidity and mortality than currently utilized strategies[7]. Furthermore, there is an important link between inflammation and poor perioperative outcomes[7,8]. Thus interventions that moderate the inflammatory response may prove to reduce adverse outcomes[9].

Many anti-inflammatory therapies have been tested in the perioperative period. The anti-inflammatory effects of corticosteroid are well established[10]. High doses of methylprednisolone or dexamethasone are important biologic modifiers of perioperative inflammatory responses in cardiac surgical patients, and reduce perioperative organ dysfunction[11]. Similarly, in non-cardiac surgery, high-dose steroids reduce the inflammatory response and improve outcomes[12,13]. Enthusiasm for use of corticosteroids in cardiac surgery has been dampened by concerns over the potential risks of such large doses. However, two major reviews concluded that a single large dose of corticosteroids appears to be harmless in the absence of specific contraindications[11,14]. Moreover, Kilger et al. observed a significant reduction in circulating inflammatory marker concentrations, along with improved outcomes after a small dose of hydrocortisone[15]. And finally, in a randomized trial of patients undergoing laparoscopic cholecystectomy, 8 mg of dexamethasone resulted in significantly lower CRP levels, significantly reduced postoperative fatigue and postoperative nausea and vomiting (PONV), and a faster return to recreational activities[16].

Another factor influencing the surgical stress response and inflammation, and thus postoperative outcomes, is anesthetic management[17]. For example, deep anesthesia may be associated with adverse outcomes including mortality. Lennmarken et al. showed that duration at deep anesthetic levels (BIS < 45) was significantly related to 1-year mortality, increasing it by 20% per hour,[2] and that non-survivors spent more time at deep BIS levels than the survivors[18]. In a cohort of adults having non-cardiac surgery under general anesthesia, lower BIS levels were independently associated with higher mortality[3]. In this study, most deaths were attributed to either cancer or cardiovascular etiologies, the pathogenesis of which has been well linked to inflammation[19-21]. The authors postulated that prolonged deep anesthesia increases mortality by aggravating the inflammatory response to surgery[3]. In support of that theory, a pilot study in orthopedic joint replacement patients demonstrated that patients who received BIS-guided anesthesia (target 45-60) showed a reduced post-operative inflammatory CRP response compared to deeper standard clinical practice[22].

Hyperglycemia is a physiologic response to surgical stress and is associated with adverse outcomes[23] such as impaired wound healing and increased infection risk. Surgical patients and those suffering acute illnesses often develop hyperglycemia, even in the absence of a preexisting diabetes[24]. Hyperglycemia is pro-inflammatory and provokes release of inflammatory cytokines. Fasting blood glucose concentration is independently related to CRP levels[25]. On the other hand, insulin *per se *is anti-inflammatory and might thus prove beneficial[26,27]. Van den Berghe et al. showed in a prospective randomized trial that intensive insulin therapy to maintain blood glucose at or below 110 mg/dL decreased inflammatory markers, and significantly reduced overall hospital mortality, blood stream infections, and acute renal failure among patients in the surgical intensive care unit (ICU)[28]. Normoglycemia also significantly reduced the use of catecholamines, and improved long-term rehabilitation[29].

The use of normoglycemia or tight glucose control is not well established in the perioperative period. In a study in cardiac surgical patients, continuous intravenous insulin infusion reduced the incidence of deep sternal wound infection and reduced mortality in diabetic patients[30,31]. But in another single-center randomized trial in a similar patient population, tight glycemic control intraoperatively did not improve outcomes,[32] although there was a relatively small difference in blood glucose concentrations in the two groups. Furthermore all patients received intensive glucose control in the ICU following their surgery, which may have lessened the effects of intraoperative glucose control[33].

The effects of intraoperative intensive vs conventional glucose control on perioperative outcomes in major non-cardiac surgery remain unknown. Normoglycemia might decrease postoperative complications such as respiratory, cardiovascular, renal and neurologic events. One outcome which has not been studied in the context of tight glucose control is postoperative delirium. Postoperative delirium is common after certain surgical procedures with a reported incidence as high as 20-60%[34]. Delirium is an important complication as it significantly impacts postoperative recovery[35]. The pathogenesis of delirium remains poorly understood, but there are reasons to believe that inflammation contributes. In vascular surgery patients, higher preoperative CRP concentrations augment the probability of postoperative delirium[35]. In hip fracture surgery, CRP concentrations were significantly greater in delirious vs. nondelirious patients [36]. And finally, intraoperative glucose concentrations were significantly greater in cardiac surgical patients experiencing a primary composite outcome that included delirium[23].

Available evidence suggests that blunting the inflammatory response to surgical trauma might improve perioperative outcomes. The putative benefits from blunting the surgical stress response are likely to be greatest in high-risk patients such as those having major non-cardiac surgery. We are thus studying three interventions potentially modulating perioperative inflammation, corticosteroids, tight glucose control and light anesthesia and their effects on major morbidity and mortality resulting from major non-cardiac surgery.

### Primary hypotheses

major perioperative morbidity in patients having major non-cardiac surgery is reduced by: 1) low-dose dexamethasone; 2) intensive intra-operative glucose control; and 3) lighter anesthesia.

### Secondary hypotheses

each intervention reduces circulating concentrations of the inflammatory marker CRP; CRP concentration correlates with post-operative complications; anesthetic sensitivity predicts major and minor complications, including delirium. Other secondary hypotheses are that each intervention reduces minor surgical complications, reduces PONV, reduces postoperative delirium, speeds hospital discharge, improves quality of life (SF-12v2 Health Survey, Christensen's VAS fatigue score), and reduces all-cause one-year mortality.

## Methods/Design

### Setting and Population

Patients scheduled for elective major non-cardiac surgeries at Cleveland Clinic will be evaluated during their preoperative anesthesia clinic visits:

#### Inclusion Criteria

1) Age ≥40 years old;

2) Major non-cardiac surgical procedures;

3) Written informed consent.

#### Exclusion Criteria

1) Recent intravenous or oral steroid therapy (within 30 days); inhaled steroids are permitted;

2) Any contraindications to the proposed interventions;

3) ASA Physical Status > 4;

*4*) Procedures done under regional anesthesia.

#### Ethics

The study protocol has been reviewed and approved by the Cleveland Clinic Institutional Review Board (IRB # 07-010)

### Study Protocol

Patients will be randomly assigned to *each *of the following interventions:

1) **Intravenous Dexamethasone or placebo**: 8 mg of dexamethasone (or placebo) will be given 1-2 hours before incision, 4 mg on the first postoperative morning, and 2 mg on the second postoperative morning.

2) **Intensive or conventional glucose management**: Patients will be randomized to blood glucose concentrations of 80-110 mg.dL^-1 ^(intensive control with a specific algorithm) or 180-200 mg.dL^-1 ^(conventional control). Glucose control will begin shortly after induction of anesthesia using pre-designed protocols and continue through the first 2 hours of postanesthesia care unit (PACU) stay. Patients will then follow the routine of the ICU/hospital ward where they will be admitted.

3) **Lighter or deeper anesthetic management**: Patients will be assigned to a target BIS of 55 (lighter anesthesia group) or 35 (deeper anesthesia group)

Randomization will be generated by a web-based system that will be accessed before anticipated induction of anesthesia, and will be stratified by history of diabetes.

Patients may be premedicated with intravenous midazolam. Prophylactic antibiotics will be given per surgical routine. General anesthesia will be induced with fentanyl and propofol. Tracheal intubation will be facilitated by succinylcholine or a non-depolarizing muscle relaxant. Additional non-depolarizing muscle relaxants will be given as necessary. Anesthesia will be maintained with sevoflurane in O_2 _and air combined with a fentanyl infusion; anesthetic drugs will be managed based on the randomized strategy; that is a BIS near 35 or 55. The lungs will be mechanically ventilated to maintain end-tidal PCO_2 _near 35 mmHg. Normothermia will be maintained with forced-air warming[37]. Blood pressure will be controlled to within a range of +20% to -30% of the preoperative baseline value. Heart rate will be controlled within a range of 40-90 beats/minute.

Red blood cell transfusions are immunosuppressive[38]. Red cell transfusions will thus be controlled by protocol. Target minimum hematocrits (HCT) will be determined based on the patient's cardiovascular status. The HCT will be maintained at 25-28% in patients without substantial cardiac disease, but maintained at 30% in those with significant cardiac disease, defined as previous myocardial infarction, angina, congestive heart failure, or cardiomyopathy.

### Measurements

We will record demographic data, ASA physical status, medical history, drug usage, preoperative hemoglobin and hematocrit, BUN and creatinine, electrolytes, and pre-operative electrocardiogram. A BIS sensor will be applied to the forehead before induction and connected to a BIS monitor. Anesthetic data will include: the volatile anesthetic dose in MAC-hours, as well as total doses of propofol and other sedative hypnotics. Distal esophageal temperature will be recorded. Blood loss will be estimated; urine output and fluid administration including allogenic blood will be recorded. Blood pressure and heart rate will be recorded. Automated intra-operative ST segment values will be recorded every 15 minutes. BIS values will be recorded electronically at one-minute intervals. Perioperative use of antibiotics and intraoperative vasoactive drugs will be recorded.

We will record the admission glucose concentration and at least hourly glucose concentrations during surgery and during the first two postoperative hours; the results of any additional glucose determinations throughout the hospital stay (obtained for clinical purposes) will also be recorded. We will use previously described methodologies to determine efficacy and safety of the insulin infusion protocols, including proportion of time spent within target range and number of hypoglycemic episodes (< 40 mg.dL^-1^)[39]. We will also measure time-weighted average (TWA) glucose and time taken to achieve desired level of glucose control. We will compare our results to other published findings in similar trials; the closest to our trial is that of Gandhi et al who investigated almost the same targets in their randomized trial in cardiac surgery patients[32]. The time to PACU discharge will be recorded, along with days in the ICU if applicable, duration of postoperative mechanical ventilation in hours, total post-operative opioid use, and the duration of hospitalization in days. Blood for high-sensitivity CRP, creatinine phospho-kinase and cardiac troponin T measurements will be sampled at the time of induction, at post-operative day 1 and 2. A 12-lead electrocardiogram will be performed immediately post-operatively and the subsequent morning.

We will use the Confusion Assessment Method (CAM) as it is the most commonly used tool in the study of delirium. It is easily performed, has a sensitivity of 94% to 100% and a specificity of 90% to 95%[40]. Christensen's fatigue VAS [41] is one of the most widely used measures of postoperative fatigue. The test will be administered pre-operatively and on post-operative days 1 and 3. An increase of three or more units on the 10- point scale will be considered a clinically significant increase.

### Data Analysis

Our primary outcome is the occurrence of at least one major complication (Table 1) in a patient within the same hospitalization and 30-day mortality. Our composite is a minor modification of the composite outcome used by Brandstrup et al.[42] and Nisanevich et al.[43]. The use of a composite adverse outcome indicator rather than independently evaluating specific complications likely reduces the chance of Type 2 error. More importantly, using any single outcome may not capture the entire effect of any of our interventions or the complex disease processes. All analyses will be intention-to-treat.

**Table 1:** Components of the composite primary outcome

Major Complications	Requirements for acceptance
Deep or organ/space surgical site infection	CDC criteria[49]
Sepsis	Positive blood culture and at least two of the following: Hypo or hyperthermia, tachycardia, tachypnea, leucopenia/leukocytosis ± DIC or multiorgan dysfunction
Bowel and surgical anastomosis stricture/obstruction or anastomotic leak	Requiring surgical intervention
Vascular graft thrombosis	Requiring surgical intervention
Bleeding	Requiring transfusion > 4 units of RBCs within the first 72 hours after surgery
Large peritoneal/pleural effusion	Diagnosed by X-Ray, ultrasound, and/or aspiration, and requiring chest tube, surgery, or ICU admission
Internal or external fistula formation	Requiring intervention
Stroke	New focal neurologic deficit of presumed vascular etiology persisted more than 24 hr with a neurologic study that did not indicate a different etiology
Pulmonary emboli (PE)	Sudden death or confirmation by V-Q scan showing high probability for PE, spiral CT scan or pulmonary arteriogram
Pulmonary edema and congestive heart failure	Shortness of breath, crepitation, peripheral edema and third heart sound and radiologic signs (cardiomegaly, interstitial edema, alveolar edema), medical treatment with diuretics
Myocardial infarction	ECG changes and/or elevated myocardial enzymes (cTn-T ≥0.2 ng/mL and/or CK ≥170IU and MB ≥5%)
Ventricular arrhythmias	ECG changes requiring medical treatment and/or electro-conversion
Renal failure	Requiring dialysis
Mortality	All-cause death within 30 postoperative days
Respiratory failure	Requiring intubation for more than 3 days
Pneumonia	New infiltrate on CXR combined with 2 of the following: temperature > 38°C, leukocytosis, and positive sputum or bronchial culture

CDC = Center for Disease Control; DIC = Disseminated Intravascular Coagulopathy; ICU = Intensive Care Unit; VQ = Ventilation Perfusion; CT = Computed Tomography; ECG = Electrocardiogram; CXR = Chest X-Ray

#### Primary outcome

Multivariable logistic regression will be used to simultaneously assess the effects of the three randomized interventions, low-dose dexamethasone (vs placebo), intensive intra-operative glucose control (vs conventional) and light anesthesia (vs deep), on the primary outcome. We will first assess the 3-way and 2-way interactions among the interventions. In absence of interactions, main effects will be assessed by collapsing over the other interventions. Otherwise, the main effects will be assessed within levels of the interacting factors.

#### CRP

A linear mixed effects model (random subject, fixed interventions and time) will be used to assess the effects of the three interventions on CRP at the 1^st ^and 2^nd ^postoperative days, adjusting for baseline. In addition, the association between CRP (each time, plus change from baseline) and outcomes of interest will be assessed using logistic regression, adjusting for intervention.

#### All-cause mortality

Multivariable Cox proportional hazard survival models will be used to assess the relationship between the interventions and all-cause mortality, comparing groups on overall and 12-month survival.

#### Additional outcomes

Time-to-event outcomes (e.g., time to death, hospital LOS, and ICU LOS) will be analyzed using Cox regression, while binary outcomes (e.g., minor perioperative complication composite (Table 2), delirium, and PONV) will be analyzed using logistic regression, and continuous variables (including SF-12) using linear regression or a non-parametric alternative. Additional analyses will be performed adjusting for any baseline potentially confounding factors for which clinical imbalance between randomized groups is observed. The significance level will be 0.05 for all main effect hypotheses, and 0.10 for interactions. Table 3 summarizes the timing of interventions and outcomes' measures.

**Table 2:** Minor Complications

Complication	Requirements for acceptance
Superficial incisional surgical site infection, or hematoma	Surgical evacuation of hematoma and or CDC
Intra-operative ST segment ischemia	An ischemic episode is defined as an ST segment change showing either ≥ 1 mm depression or ≥ 1.5 mm elevation from baseline
Unplanned ICU admission	Unplanned ICU admission
Minor surgical intervention	Surgeon called for care issues during the first 2 hours postoperatively
Non-ventricular arrhythmias	ECG changes, medical Rx and/or electroconversion
Small peritoneal/pleural effusion	Diagnosed by X-Ray, ultrasound, and/or aspiration, and not requiring chest tube, surgery, or ICU admission
Ileus	Lasting more than 72 hours
DVT	Diagnosed by Doppler examination, venogram, or CT scan
Cystitis or urinary tract infection	Fever, dysuria and positive urine culture
Hemodynamic disturbances	Requiring vasoactive drugs and/or β blocker treatment in the first 2 hours postoperatively
Progressive renal insufficiency	Rise in creatinine of > 2 mg/dl from pre-operative value but with no requirement for dialysis

CDC = Center for Disease Control; ICU = Intensive Care Unit; DVT = Deep Venous Thrombosis; Rx = treatment; ECG = Electrocardiogram; CT = Computed Tomography

**Table 3:** Flow -chart of timing of the interventions and outcomes measures

	Pre-op visit	Preop Immediate	Intra-op	Post-op immediately	POD1	POD2	POD3	Hospitalization	30-day	6, 12 months
		Dexamethasone (8 mg)			Dexamethasone (4 mg)	Dexamethasone (2 mg)				
	
**Interventions**			Depth of Anesthesia							
	
			Glucose Control	Glucose Control						

										

								Composite Primary outcome	Primary outcome (mortality)	
	
								Secondary outcomes	Secondary outcomes	Secondary outcomes
	
	SF-12								SF-12	
	
**Outcomes**	Christensen Fatigue Score				Christensen Fatigue Score		Christensen Fatigue Score			
	
					CAM ICU - twice daily	CAM ICU - twice daily	CAM ICU - twice daily			
	
									Phone call- vital status	Phone call- vital status
	
	EKG			EKG	EKG					
	
				Cardiac Enzymes 3 × 8 hrs apart						
	
		CRP			CRP	CRP				

POD = post operative day; SF 12 = health-related quality of life measure; CRP = C-Reactive Protein; CAM ICU =Confusion Assessment Method-ICU

### Interim Monitoring Plan and Adaptive Design Options

The first stage of the study will be a traditional (i.e., non-adaptive) group sequential design[44,45], where interim analyses for both efficacy and futility will be conducted after 25%, 50% and 75% of the patients have been enrolled, and at the end of planned enrollment, as needed. Interventions with either a large or very small treatment effect will likely cross boundaries for efficacy or futility, respectively, at one of the interim analyses, and be stopped early. By the final analysis (including a possible adaptive stage, as explained below), all of the interventions will have either crossed an efficacy or futility boundary.

#### Interim Monitoring Details

**Figure 1:**
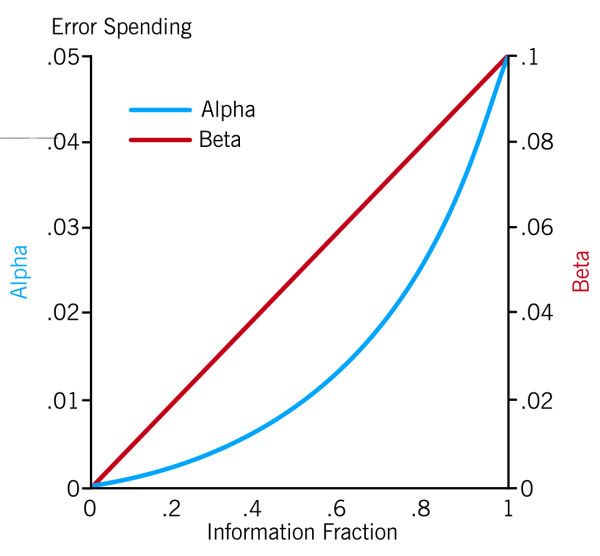
**Spending functions for alpha or type I error (on left vertical axis) and beta or type II error (on right vertical axis) as a function of information time on horizontal axis, where information time is the proportion of the maximum planned sample size available for a particular interim analysis. **Lines represent the cumulative error spent throughout the trial. Beta is spent faster than alpha to allow the study to stop early more readily for futility than for efficacy. Lines are continuous, allowing flexibility in choice of monitoring times, but interim monitoring will only be done at the pre-specified times for this study.

**Figure 2:**
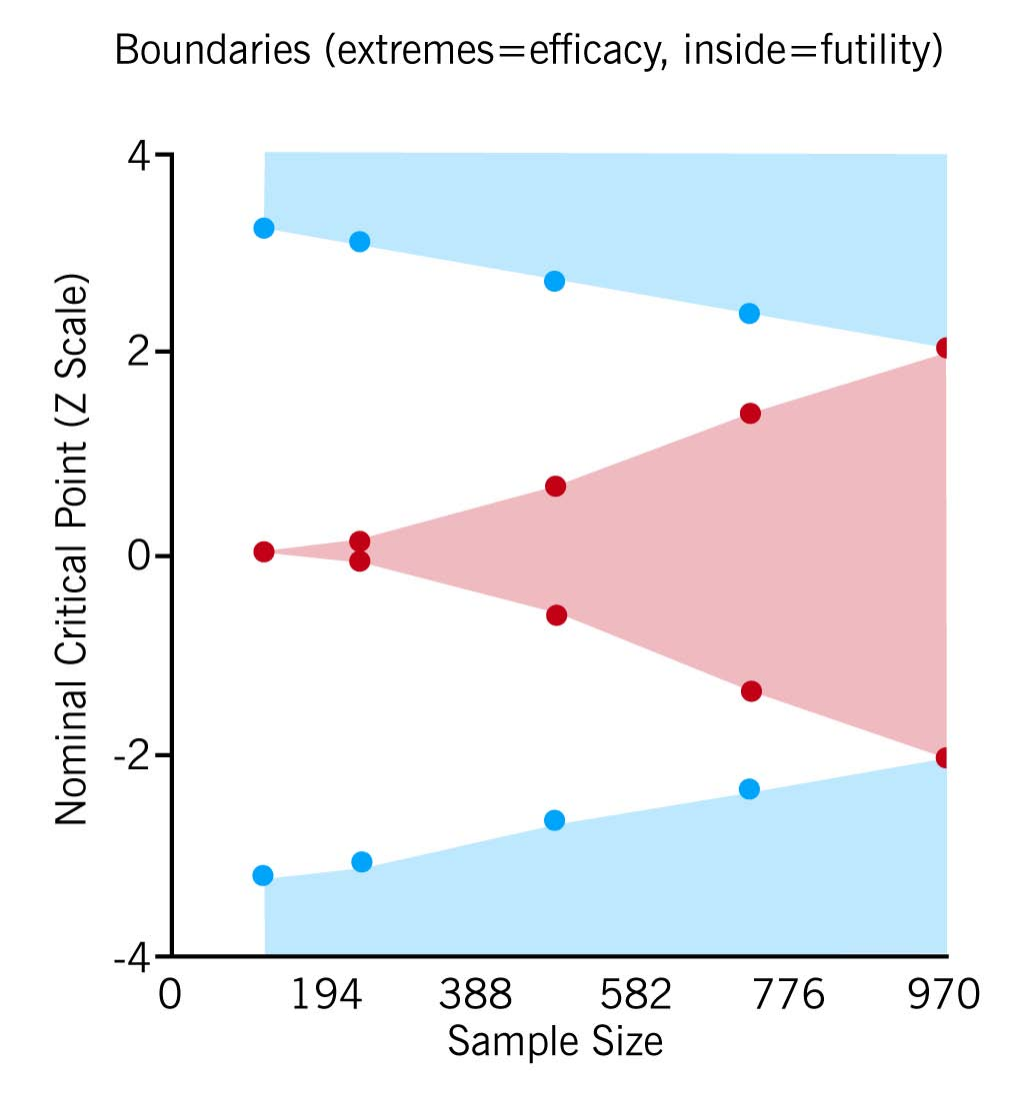
**Stopping boundaries for efficacy and futility. Plot of the standardized treatment effect (z- statistic) on vertical axis and accrued sample size on the horizontal axis, where a z-statistic of zero indicates no treatment effect. **Outside shaded regions indicate sufficient evidence to reject null hypothesis for efficacy (top) or harm (below), while inside shaded region indicates evidence to stop for futility, or no effect.

We will use the gamma spending function[46] to monitor efficacy (gamma = -3, similar to O'Brien-Fleming) and futility (gamma = 0, similar to Pocock), spending Type I error, which monitors efficacy, slower than the Type II error, which monitors futility, in order to facilitate early stopping for either, but more so for futility (Figure 1). P-values for crossing efficacy (futility) boundaries for the 1^st^, 2^nd^, 3^rd ^and final analyses will be ≤ 0.003 (> 0.972), ≤ 0.007 (> 0.936), ≤ 0.017 (> 0.526) and ≤ 0.041 (> 0.041), respectively, for the 3 interim looks and final analysis of the initial group sequential stage (Figure 2). The overall significance level of 0.05 and power of 0.90 will be maintained. Interim results, along with a recommendation on whether to continue any or all interventions, will be given to the study's Executive Committee which will determine whether to halt or continue the trial at each juncture.

#### Adaptive Design Option

At the third planned group sequential analysis (N = 728 patients), an adaptive sample size extension of the trial may be proposed to study one or more of the interventions that showed a modest but important treatment effect but had not yet crossed an efficacy (or futility) boundary. The method of Muller and Schafer[47] would be used to the extent that intervention with an increased sample size in a second stage while preserving the overall Type I error of the study. A second stage with new stopping boundaries would be designed with sample size and planned interim analyses to be able to detect a revised treatment effect with the desired power. Results from the 1^st ^and 2^nd ^stages for that intervention would be combined in a final analysis. East and SAS statistical software will be used for the sample size calculations, interim analyses and final analyses.

### Sample size considerations

We estimate that 25% of patients receiving no intervention (i.e., the control) will have at least one major complication. We further hypothesize that all interventions will not have the same effect and will power the study for a relative reduction of 40% or more for at least one intervention, and 20% and 10% versus respective controls for the other two. Our design will thus require a maximum of 970 total patients for the original group sequential portion of the study to have 90% power at the 0.05 significance level to detect a 40% reduction on the primary outcome for the most promising intervention.

## Discussion

A factorial design will enable us to simultaneously study the effects of the three interventions; dexamethasone vs placebo, intraoperative tight vs. conventional glucose control, and light vs deep anesthesia, in the same population, both individually and in different combinations. Such a design is an economically efficient way to study the three interventions in one clinical trial vs three, especially if there is no statistical interaction between the treatments[48].

In fact, we do not expect an interaction between the three interventions; that is, the effect of one intervention should not depend on the presence or absence of the other. But in the event that the efficacy of one intervention does depend on another (*e.g.*, steroids improve outcomes only if glucose is controlled), a factorial design will enable us to quantify the magnitude of the interaction. In contrast, this sort of interaction would be impossible to observe with three separate univariate trials. Factorial design is thus not only more efficient, but often superior to conventional trials that evaluate only a single intervention.

In our case, all three interventions are presumed to exert their protective effects through the same mechanism -- modulation of the peri-operative inflammatory response -- albeit possibly *via *different pathways. However, the putative effects of each are limited by a ceiling effect. Thus, a strength of the proposed factorial design is that we will be able to evaluate the presumed additive benefits of each intervention.

An added strength to our trial is that we will evaluate "hard" outcomes (major complications and mortality) rather than intermediate or indicator outcomes. Furthermore, the interventions we will test are fairly easy to use, inexpensive, and low-risk.

### Limitations

The dose of steroids we have chosen may prove sub-optimal. Large-dose steroids have been shown to improve perioperative outcomes in patients undergoing cardiac or colorectal surgery[11,14]. However, concerns were raised about the possibility of theoretical side effects of such a large dose. Subsequent work by Kilger and associates suggests that much smaller doses are also effective,[15] and are -- presumably -- considerably safer. The anti-inflammatory potency of the dose we have chosen is comparable to that used by Kilger, although, we used dexamethasone rather than hydrocortisone[49]. It is also in line with the study by Bisgaard et al. that resulted in significantly lower CRP levels and improved recovery parameters. There was no increase in wound infection or other adverse outcomes[16]. Steroid administration will start 1-2 hours before surgery because the effects of steroids are believed to be mediated by protein synthesis which usually takes an hour or two[50].

Patients will be randomized to either a tight glucose control group with a goal of 80-110 mg·dl^-1 ^or to a conventional care group with a goal of 180-200 mg·dl^-1^. The lower range is that used by Van den Berghe et al, [28] and was shown to be beneficial in critical care patients. There were few complications associated with tight control in such a low range. The higher range essentially corresponds to current routine clinical practice. For example, a recent retrospective study showed that the rates of insulin treatment among academic anesthesiologists for glucose values < 140 mg·dl^-1^, 140-200 mg·dl^-1^, or > 200 mg·dl^-1 ^were 0.1%, 1.4%, and 11.9%, confirming that many anesthesiologists do not treat intraoperative glucose values less than 200 mg·dl^-1^[51].

Clinicians and study coordinators will be blinded to dexamethasone treatment. But due to the nature of the interventions, clinicians will not be blinded to the randomization regarding level of glucose control and depth of anesthesia in any given patient. The data collectors for postoperative events will be blinded to all three interventions.

### Conclusion

The DeLiT Trial is a multi-factorial randomized single-center trial of dexamethasone vs placebo, intraoperative tight vs. conventional glucose control, and light vs deep anesthesia in patients undergoing major non-cardiac surgery. The primary outcome is a composite of major post-operative morbidity including myocardial infarction, stroke, sepsis, and 30-day mortality. C-Reactive protein, a measure of the inflammatory response, will be evaluated as a secondary outcome. One-year all-cause mortality as well as post-operative delirium will be additional secondary outcomes. We will enroll up to 970 patients which will provide 90% power to detect a 40% reduction for the primary outcome, including three equally spaced interim analyses for efficacy and futility.

## List of abbreviations

ASA: American Society of Anesthesiologists; BIS: bispectral index; BUN: blood, urea, nitrogen; CAM: confusion assessment method; CDC: Center for Disease Control; CRP: C-Reactive protein; CT: Computed Tomography; CXR: chest x-ray; DIC: Disseminated Intravascular Coagulopathy; DVT: deep vein thrombosis; ECG: electrocardiogram; HCT: hematocrit; ICU: intensive care unit; LOS: length of stay; MAC: minimum alveolar concentration; PACU: postanesthesia care unit; POD: postoperative day; PONV: postoperative nausea and vomiting; RCT: Randomized Controlled Trial; Rx: Treatment; SF 12: health-related quality of life measure; TWA: time weighted average; VAS: visual analogue scale; VQ: Ventilation perfusion;

## Competing interests

Financial disclosure:

BA: Received research funding from Aspect Medical (currently Covedien), and Hutchinson Inc. AM: EM, AK TM, SS, DS: 'These author declare that they have no competing interests' WT: Abbott Laboratories Research Funding. TS: 'This contributor declares that she has no competing interests'.

## Authors' contributions

BA: PI participated in study concept and design, study conduct, data analysis, manuscript writing. AM: study coordinator, participated in study conduct, patients' consenting, recruiting. EM: Senior Study Biostatistician, participated in study design, statistical analysis plan, sample size calculations and manuscript writing. SS: participated in the study design and conduct/recruiting and manuscript review. TM: participated in study conduct, manuscript review. WT: Co-investigator, participated in the biomarkers testing and analysis and manuscript review. AK: participated in study design, manuscript review and editing. DS: Senior investigator; participated in study concept and design, data analysis manuscript writing, corresponding author.

All authors read and approved the final manuscript

## Pre-publication history

The pre-publication history for this paper can be accessed here:

http://www.biomedcentral.com/1471-2253/10/11/prepub
